# Supplementary figures and images for: Quantification of hypoxic regions distant from occlusions in cerebral penetrating arteriole trees
Source: PLoS Comput Biol. 2022 Aug 5;18(8):e1010166. doi: 10.1371/journal.pcbi.1010166 (PMC9385041; doi:10.1371/journal.pcbi.1010166)

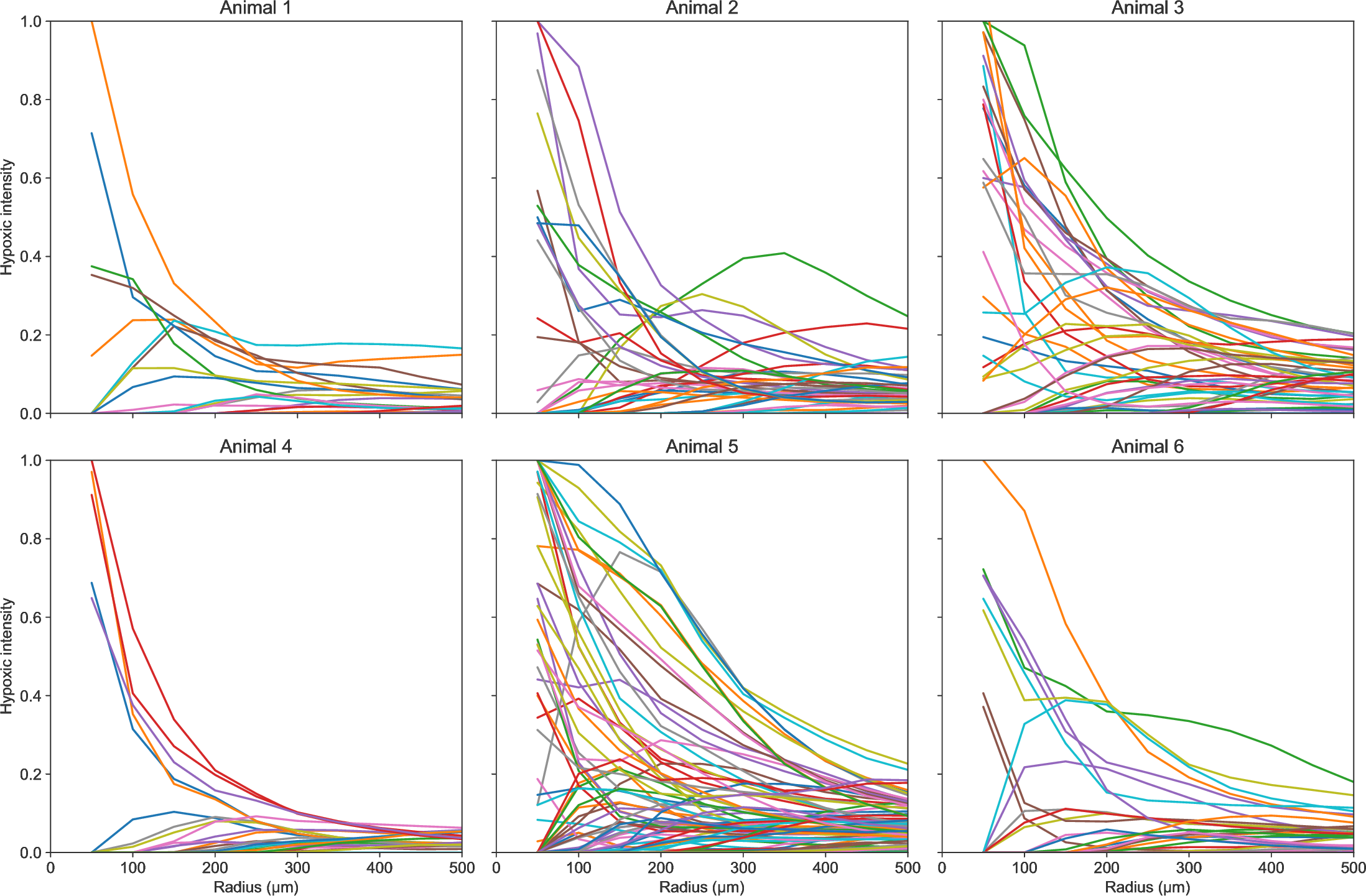

Supplement: S1 Fig — (TIF) [file pcbi.1010166.s001.tif]

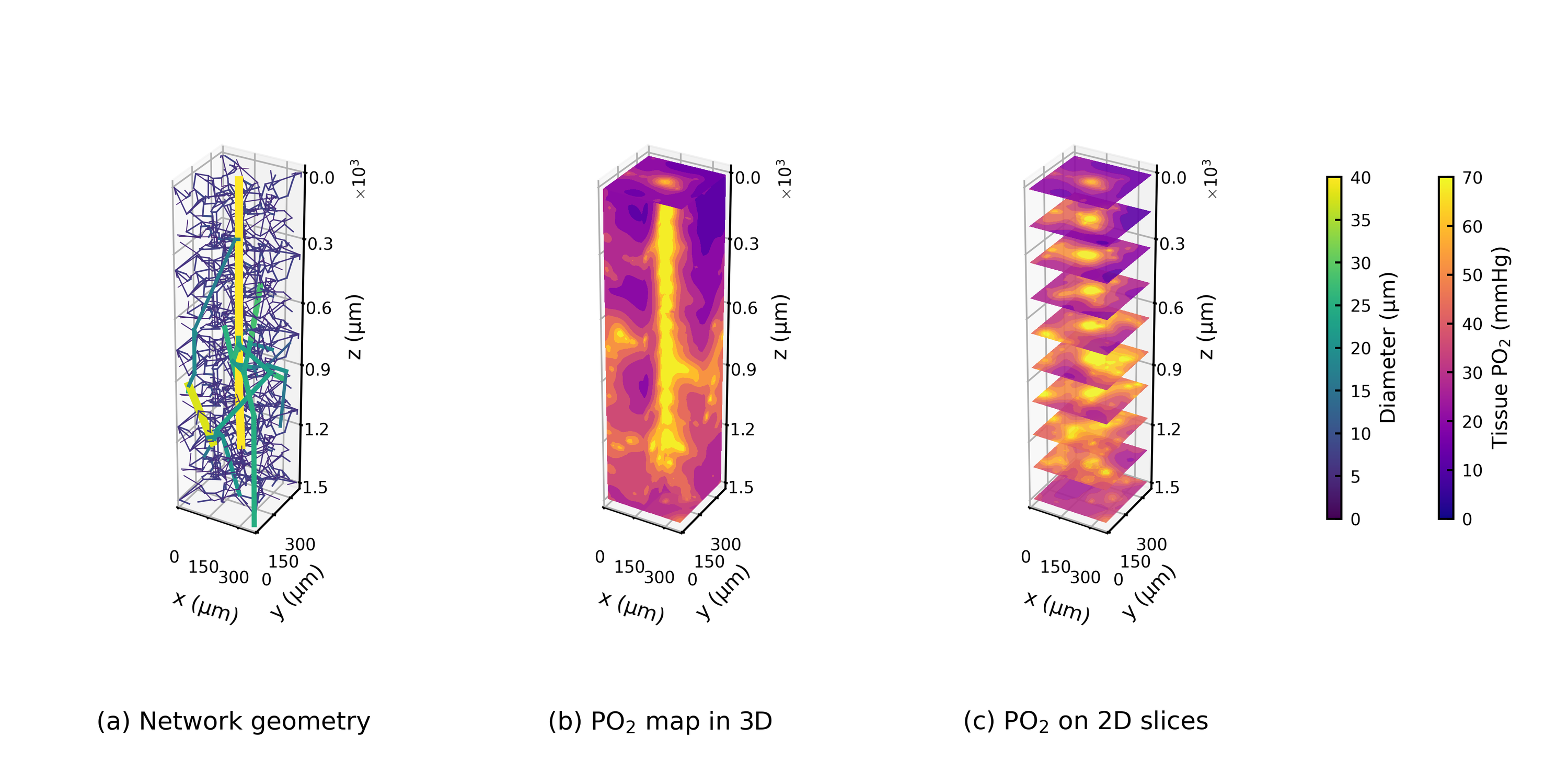

Supplement: S2 Fig — The geometry of a column with the same arteriole geometry as shown in Fig 7A but different capillary geometry (a) and its tissue oxygenation (b, c). (TIF) [file pcbi.1010166.s002.tif]

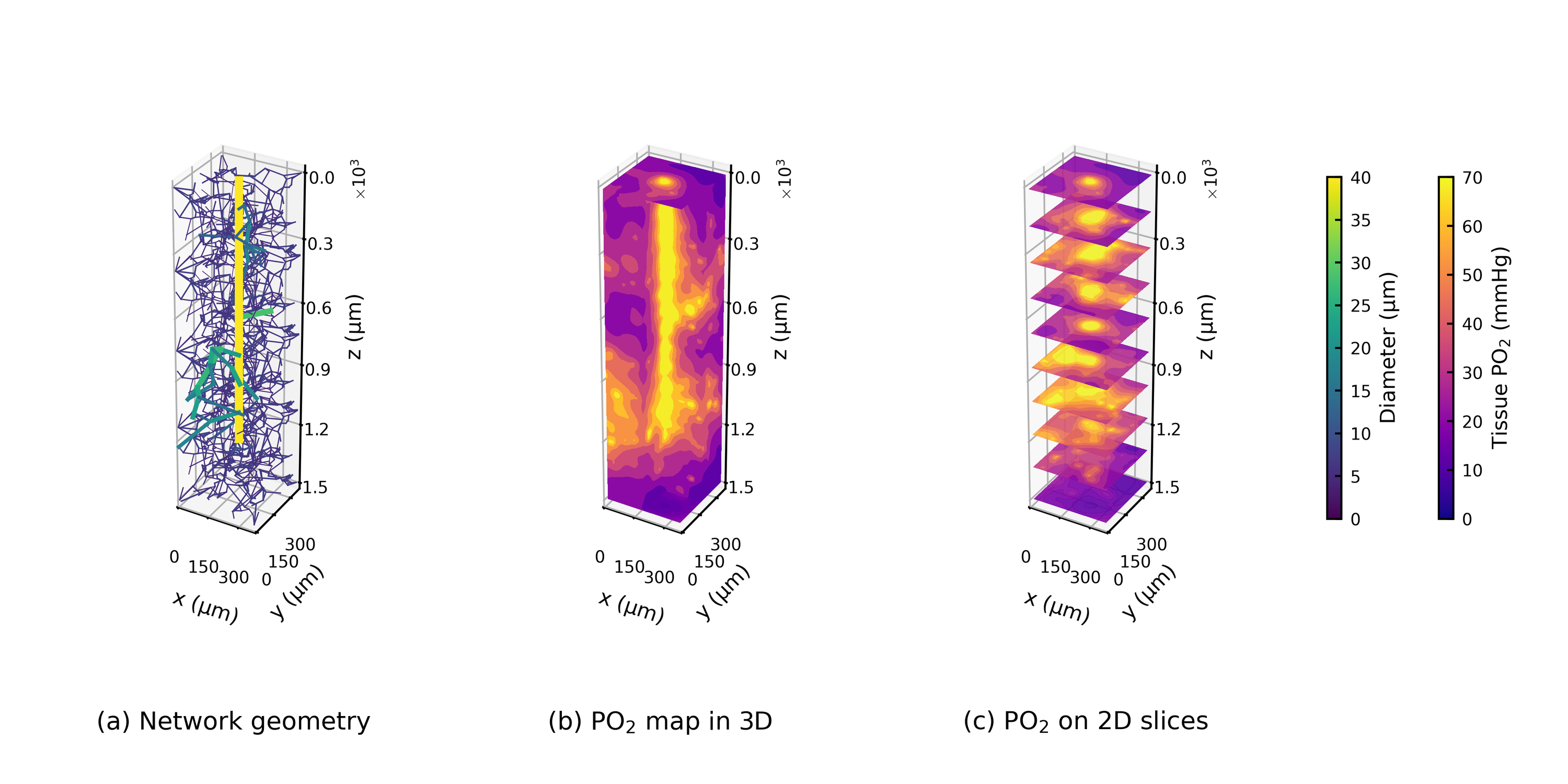

Supplement: S3 Fig — The geometry of a column with the same capillary geometry as shown in Fig 7A but different arteriole geometry (a) and its tissue oxygenation (b, c). (TIF) [file pcbi.1010166.s003.tif]

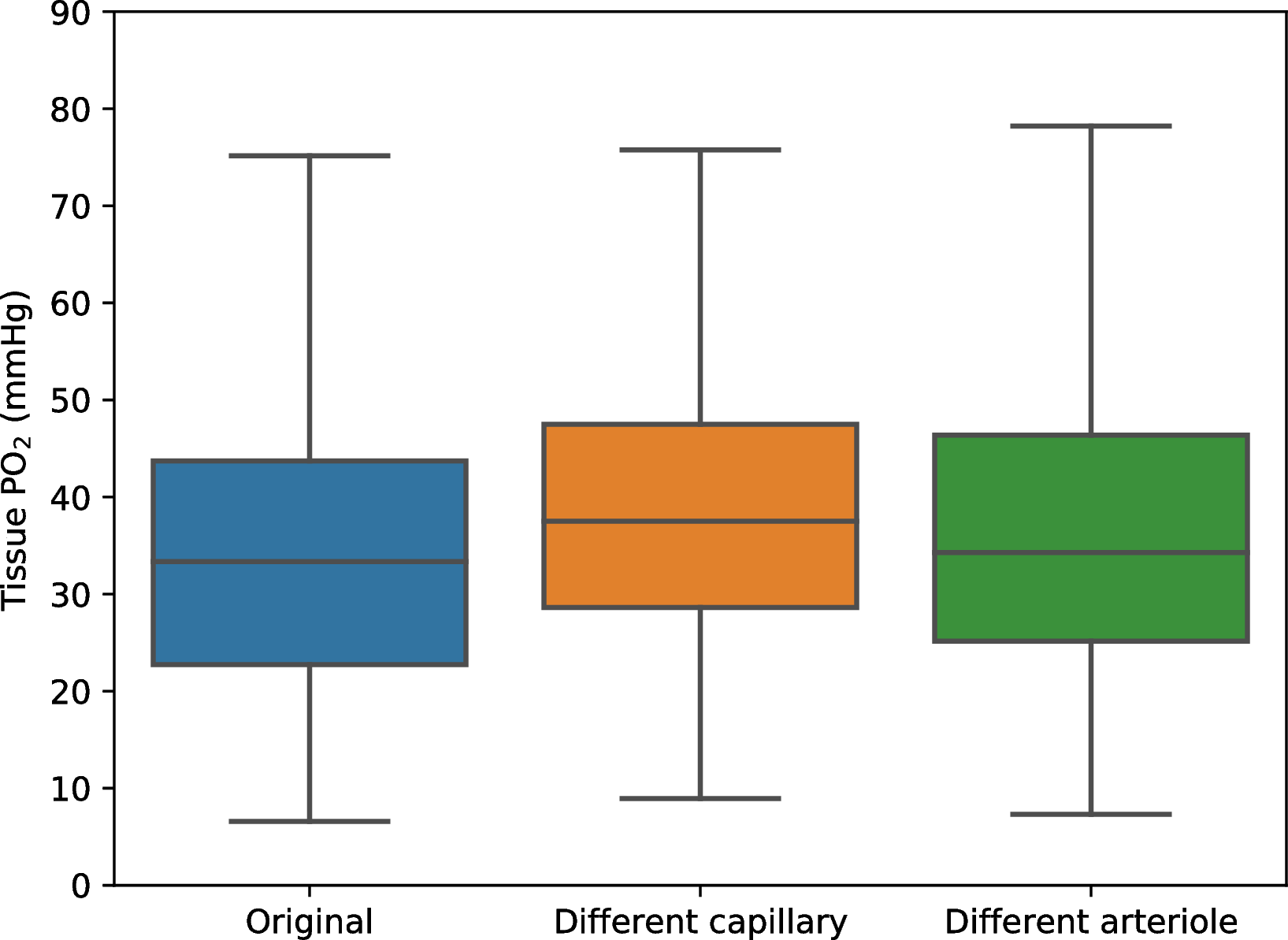

Supplement: S4 Fig — Tissue PO2 distribution in cortical columns shown in Figs 7 and S2 (using a different capillary cube) and S3 (using a different penetrating arteriole tree). (TIF) [file pcbi.1010166.s004.tif]

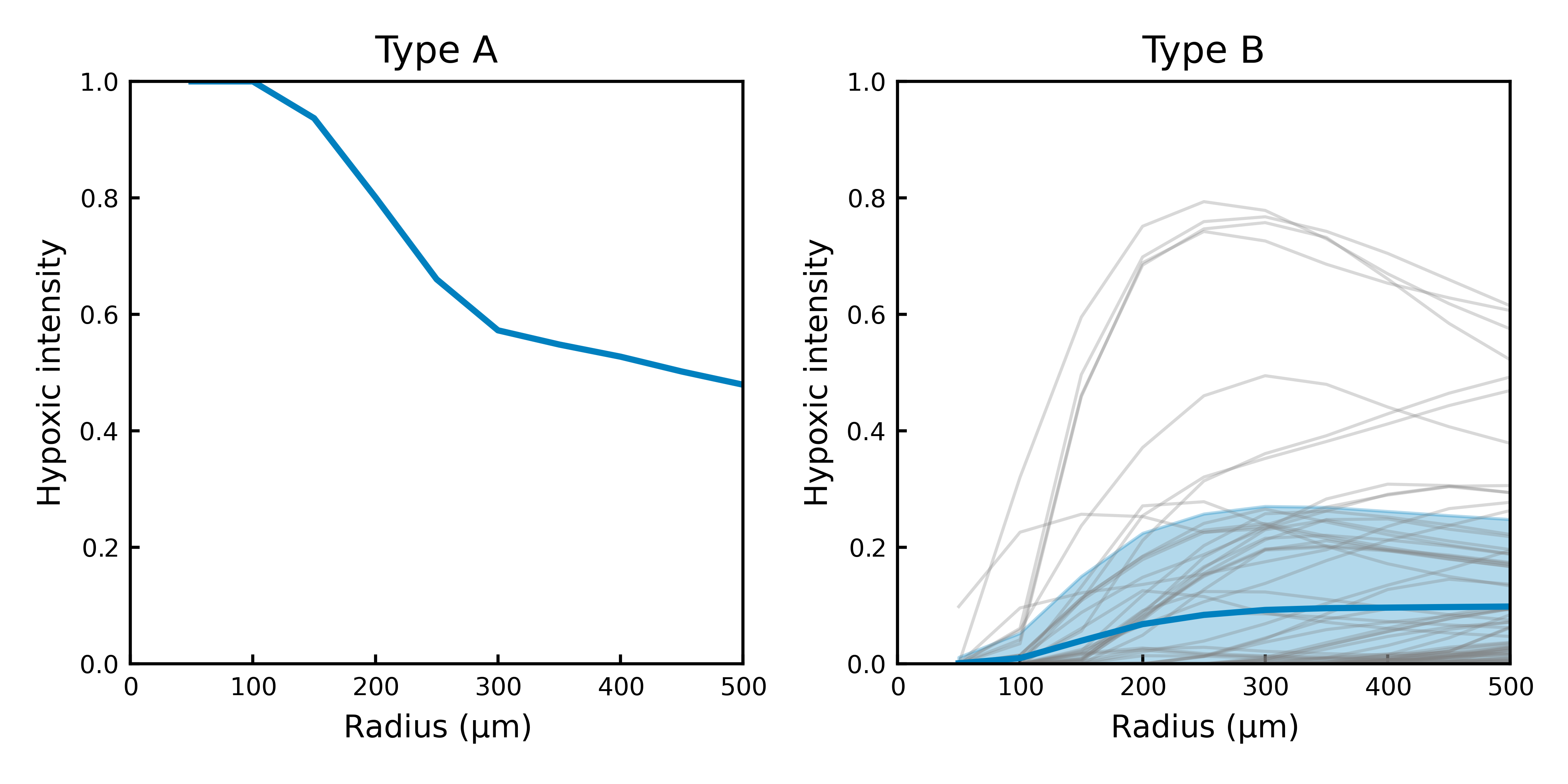

Supplement: S5 Fig — (TIF) [file pcbi.1010166.s005.tif]
