# Supplementary material for: Quantification of hypoxic regions distant from occlusions in cerebral penetrating arteriole trees
Source: PLoS Comput Biol. 2022 Aug 5;18(8):e1010166. doi: 10.1371/journal.pcbi.1010166 (PMC9385041; doi:10.1371/journal.pcbi.1010166)
Supplement: Attachment — Submitted filename: Review_response.docx [file pcbi.1010166.s007.docx]

**Detailed response for Reviewer 1’s comments:**

*Reviewer #1: The authors present experimental and theoretical results concerning the effect of local occlusion in brain cortex microvasculature on the occurrence of hypoxic regions. This is a medically important topic, because of the damage to brain tissue that can result from microemboli, such as result from breaking up larger thrombi. The results are analyzed in terms of the distribution of distances between occlusion sites and the resulting hypoxic sites. The results indicate some similarities and some differences between the theoretical simulations and the experimental results. The reasons for the discrepancies are not entirely clear, but different distributions of blockage sites through the cortical thickness appear to be a factor. Both the experimental and theoretical aspects of the work are carefully done and clearly presented. Overall, this is a novel approach to an important topic, and opens up new directions for future work on the relationship between local flow blockages and tissue hypoxia/ischemia. Consequently, this is a valuable contribution, even though the work does not lead to definitive conclusions on this topic.*

*Specific Comments:
1. Line 321. “pixel-based Gx function” The significance of “Gx” is not explained. This term could be removed without causing any ambiguity.*

**Response: Thank you very much for your comments. As mentioned in the second paragraph of Section 2.3, the term “Gx” comes from our previous study (Georgakopoulou et al., 2021, Journal of Biomechanics, reference 41), where the Gx function is defined as the cumulative density function of the nearest neighbour distances (from hypoxic regions to microspheres). In the previous study, the centre locations of hypoxic regions were used, however, we used the imaging pixels in this study. Hence, the new distance analysis has been named as the “pixel-based Gx function” to distinguish it from the GX function (see lines 329 ff).**

*2. Line 321. It would be helpful to add a comment about the purpose of the “distance analysis,” e.g. to establish spatial relationships between vessel blockages and hypoxic regions.*

**Response: Thank you very much. The purpose of the distance analyses was added as suggested (see lines 322 ff).**

*3. Line 354: should be “number and density of microspheres”*

**Response: Thank you very much. We have improved the sentence as suggested (see line 361).**

*4. Figure 4. The locations of the microspheres are virtually invisible. I suggest adding arrows or circles to show where they are.*

**Response: Many thanks for the suggestion. We have added white circle for each microsphere in Figure 4. The figure caption has also been modified accordingly.**

*5. Line 530. “By comparison, the latter one zooms out…” In principle, the two measures are giving the same information. If f(r) is the hypoxic intensity, then the cumulative distribution is the normalized value of integral(0 to r) (f(r) r^2 dr), which is approximately the pixel-based Gx function.*

**Response: Thank you very much for the comment. Yes, the pixel-based Gx function and hypoxic intensity are related to each other within a certain length scale (500 microns in this manuscript). However, the hypoxic intensity does not include the hypoxic regions further away than the maximum sphere radius. As shown in Figure 9, there are more than 20% of hypoxic regions that are 500 microns away from the occlusion sites (the pixel-based Gx function only reaches 60-80% at 500 microns). Hence, we can only know the distribution of these distant hypoxic regions by using the pixel-based Gx function. In addition, the hypoxic intensity was calculated around each microsphere, the pixel-based Gx function however was calculated as the cumulative distribution of distances for all microspheres in an animal. The pixel-based Gx function is thus also weighted by the hypoxic volume caused by each microsphere. There are therefore some subtle differences between the two measures.**

*6. Line 537. “Excellent agreement” is an overstatement. If there was excellent agreement, then the “hypoxic intensity” results could not differ by so much (see previous comment). I would say “fair agreement.”*

**Response: Many thanks for the comment. We have modified the sentence as suggested (see line 546).**

*7. Line 878: Reference seems to be incomplete.*

**Response: Thais reference is a DPhil thesis at the University of Oxford. We have added this additional information for reference 53 (see line 888).**

**Detailed response for Reviewer 2’s comments:**

*Reviewer #2: This work uses animal experiments and computational modeling to improve understanding of how embolisms in brain microvessels create both ischemia and hypoxia, and in particular how the resulting hypoxia can be either co-localized with the embolism or relatively distant from it. I feel the work is of high quality and novel, and therefore generally suitable for PLOS Computational Biology. I do have several comments I feel the authors should consider.*

*1. Although the meaning of the hypoxia-embolism distance measure Gx is fairly clear, an exact definition is not given. I feel this would be helpful in interpreting results presented (e.g., delving deeper into apparent similarities between model and experiment in Fig.9a-c), and for comparisons to other work in the future. This could be given in the main part of the manuscript or in Supporting Information.*

**Response: Thank you very much for your comment. We have added an equation to define the pixel-based Gx function in Section 2.3 (see lines 329 ff).**

*2. The hypoxia intensity measure used gives the volume fraction of hypoxia in spheres of increasing radii around an embolism (in this case, 25micron sphere). This provides very useful information, but did the authors consider using a differential measure instead, where at each radius the hypoxic density in the corresponding spherical shell is given? This would seem to have potential to give more details on the average spatial distribution of hypoxia, and possibly give a more sensitive comparison between experiment and modeling.*

**Response: This is an interesting suggestion, but after some thought, we believe that this other measure would simply end up providing the same information (and we believe that the readers can get that plot from our current hypoxic intensity plots).**

*3. It is somewhat surprising how dominated by diffusion from arterioles the calculated tissue PO2 distributions are. Has this effect been verified by comparisons to brain PO2 measurements, and has the intravascular resistance to O2 transport inside arterioles been considered in the model?*

**Response: The intravascular resistance has been considered in the model by using Fick’s law. The details of the implementation can be found in Secomb et al. (2004, reference 32) and the model was originally presented in Hellums et al., 1995, Annals of Biomedical Engineering. We didn’t measure the tissue PO2 in our experiments, but a detailed simulation and validation of our methodology can be found in Hartung et al. (2021, PLOS Computational Biology, reference 39). The penetrating arterioles have also been found to play a significant role in oxygen transport as presented in Vovenko (1999, European Journal of Physiology) and Sakadzic et al. (2014, Nature Communications).**

*4. Can the authors explain the fact that although only 31% of experimental measurements give type A hypoxic behavior (localized near the embolism), the overall effect (Fig.9d) is dominated by type A? (Note that the Fig.9 caption is not fully correct.). This seems to be a relative magnitude effect, so perhaps some other type of averaging could be considered.*

**Response: Thank you very much for the comment. The error bars of Figure 9 didn’t present correctly in our first manuscript, and these have been corrected in the revised manuscript. As shown in Figure S1, type A hypoxic intensity can have a very high starting value (close to 100%) and a sharp decrease from 50 to 100 microns. Hence the average results in a type A trend, although only about 31% of hypoxic intensity is type A (of course, different weighted averages would give different results here).**

*5. It is very interesting that in simulations almost 99% of the hypoxic behavior is type B (vs 69% experimentally). It seems the model could easily be used to test the idea that blockages close to a large vessel (ie penetrating arteriole) give type B behavior by using microspheres of decreasing size (20, 15, 10 microns), and looking for a B to A transition. I definitely suggest this approach as it would also address the possibility that 25micron spheres are blocking slightly smaller vessels experimentally.*

**Response: To address this comment, we have conducted 34 more simulations by using 15-micron microspheres in a cortical column (the one shown in Fig. 7), where one occlusion scenario leads to type A hypoxic intensity (2.9%) and 33 scenarios lead to type B (97.1%). However, the 25-micron microspheres will lead to 6 scenarios of type B (100%) in the same cortical column. Hence there is a transition from B to A when using microspheres of decreasing size. However, only using smaller microspheres cannot fully explain the 31% of type A hypoxic intensity that are shown in the experiments. As presented in the Discussion section, there are other factors including geometrical differences of both morphometry and length scale, *in vivo* active responses and pathological processes (e.g., infarct formation). As discussed by the reviewer, these hypotheses need further investigations beyond the current study.**

*6. A further test of ideas in the previous comment that the authors could consider is determining the actual size of the vessels blocked experimentally, and also the distance of each microsphere from the nearest penetrating arteriole. Even one of these pieces of information, which I think should be possible to extract from the existing dataset, would help to explain the A/B differences shown in Fig.9, and improve understanding of how and where micro-embolisms form.*

**Response: The reviewer asks A) the actual size of the vessels blocked experimentally and B) the distance of each microsphere from the nearest penetrating arteriole. The current technique of brain tissue reconstruction based on the alignment of 10 consecutive coronal sections is not appropriate to allow the extraction of geometrical data from the cerebral vasculature. Due to tissue fixation and sectioning of the brain tissue, the alignment becomes challenging, especially of the microvasculature. In a future study, we aim to extract vascular geometry data from transparent brain tissue (made with chemical tissue clearing). In addition, fixation of the brain tissue with paraformaldehyde (PFA), as done in the current study, causes tissue to shrink (Korogod et al., 2015, eLife). As a result, the calculation of the actual size of blocked vessels is not accurate at this stage but should become so in our future work.**
